# Development and Validation of a Stability-Indicating RP-UPLC Method for the Estimation of Impurities in Cinacalcet Hydrochloride API and its Formulation

**DOI:** 10.3797/scipharm.1502-06

**Published:** 2015-04-16

**Authors:** Pingili Sunil Reddy, Thummala Veera Raghava Raju, Penmetsa Satyanarayana Raju, Nadimpalli Sunil Varma, Kondra Sudhakar Babu

**Affiliations:** 1 Analytical Research and Development, Integrated Product Development, Dr. Reddy’s Laboratories Ltd., Bachupally, Hyderabad-500 072, India; 2 Srikrishnadevaraya University, Anantapur-515 055, A.P., India

**Keywords:** Cinacalcet Hydrochloride, RP-UPLC, Stability-indicating, Impurities, ICH guidelines

## Abstract

A sensitive, stability-indicating, gradient reversed-phase ultra-performance liquid chromatography method has been developed for the quantitative estimation of cinacalcet hydrochloride impurities in active pharmaceutical ingredients and pharmaceutical formulations. Efficient chromatographic separation was achieved on an Acquity BEH Shield RP18, 100 × 2.1 mm, 1.7 µm column with the mobile phase containing pH 6.6 phosphate buffer and acetonitrile. The flow rate of the mobile phase was 0.3 mL min^−1^ with a column temperature of 35°C and detection wavelength at 223 nm. The relative response factor values of (+)-*R*-1-(1-Naphthyl)ethylamine, regioisomer, diastereomer isomer-1, and diastereomer isomer-2 were 1.79, 0.99, 0.89, and 0.88, respectively. The cinacalcet hydrochloride formulation sample was subjected to the stress conditions of acid, base, oxidative, hydrolytic, thermal, humidity, and photolytic degradation. Cinacalcet hydrochloride was found to degrade significantly under the peroxide stress conditions. The degradation products were well-resolved from cinacalcet hydrochloride and its impurities. The peak purity test results confirmed that the cinacalcet hydrochloride peak was homogenous in all stress samples and the mass balance was found to be more than 96%, thus proving the stability-indicating power of the method. The developed method was validated according to ICH guidelines.

## Introduction

**Fig. 1:**
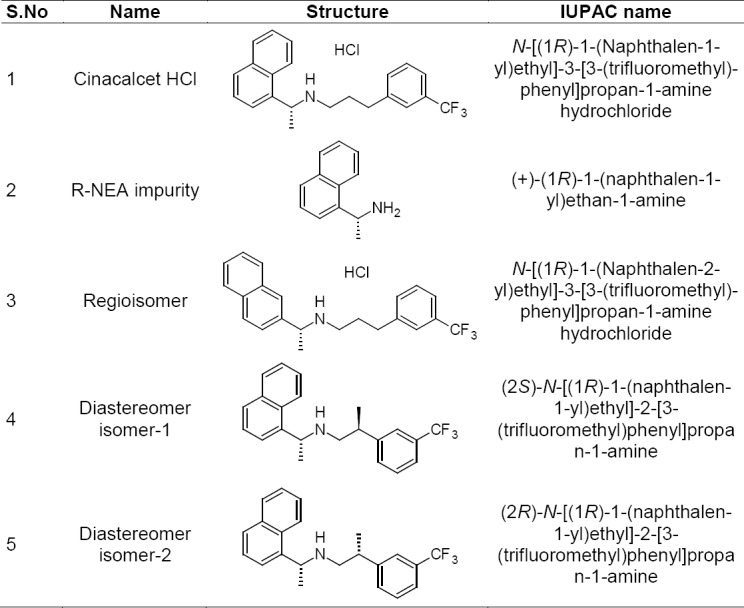
Chemical structure of cinacalcet HCl and its impurities

Cinacalcet hydrochloride (CH) is an oral calcimimetic indicated for the treatment of secondary hyperparathyroidism (HPT) in patients on dialysis with end-stage renal disease (ESRD), and in patients with parathyroid carcinoma to reduce hypercalcemia. CH is the first of a new class of drugs, the calcimimetics, which act by increasing the sensitivity of calcium-sensing receptors in the parathyroid gland [1–8]. Chemically, CH is *N*-[(1*R*)-1-(Naphthalen-1-yl)ethyl]-3-[3-(trifluoromethyl)phenyl]propan-1-amine hydrochloride (Fig. 1).

Impurity profiling of active pharmaceutical ingredients (API) and pharmaceutical formulations is one of the most challenging tasks of pharmaceutical analytical chemists under industrial environments [9]. The presence of unwanted, or in certain cases unknown chemicals, even in small amounts, may influence not only the therapeutic efficacy, but also the safety of the pharmaceutical products [10]. For these reasons, International Conference Harmonization guidelines have established maximum allowed limits for related compounds for both bulk and formulated APIs. As per the requirements of various regulatory authorities, the impurity profile study of drug substances and drug products has to be carried out using a suitable analytical method in the final product [11, 12].

A detailed literature survey revealed that there are some analytical methods reported for the estimation of CH either individually or in combination with other drugs like HPLC, spectrophotometry, and LCMS [13–18]. The route of synthesis of cinacalcet resulted in four known impurities: (+)-*R*-1-(1-Naphthyl)ethylamine (RNEA), regioisomer, diastereomer isomer-1, and diastereomer isomer-2, which are not reported in any of the pharmacopeia. Among these impurities, RNEA is the starting material, regioisomer is the intermediate, and diastereomer isomer-1 and diastereomer isomer-2 are process-related impurities during synthesis.

Hence, an attempt has been made to develop an accurate, rapid, specific, and reproducible method for the determination of CH impurities (Fig. 1) in API and in pharmaceutical dosage forms along with method validation as per ICH guidelines [19, 20]. Using the developed method, the stability of both drug substance and drug product can be evaluated as per ICH guidelines [21, 22].

## Experimental

### Chemicals, Reagents, and Samples

CH tablets were received from the formulation research and development laboratory of Dr. Reddy’s Laboratories Ltd., IPDO, Hyderabad, India. CH API and impurities were procured from Dr. Reddy’s Laboratories Ltd., CTO 6, India. Analytical grade potassium dihydrogen orthophosphate was procured from Merck, Germany. HPLC grade acetonitrile, triethyl amine, and orthophosphoric acid were purchased from Merck, Germany, and high-purity water was prepared by using the Millipore Milli-Q Plus purification system.

### Equipment

The LC system used for method development and method validation was the Waters Acquity UPLC. The output signal was monitored and processed using Waters Empower software. Weighing was performed with a Mettler XS 205 Dual Range (Mettler-Toledo GmbH, Greifensee, Switzerland). Photostability studies were carried out in a photostability chamber (SUN TEST XLS+, Atlas, USA). Thermal stability studies were performed in a dry air oven (Merck Pharmatech, Hyderabad, India).

### Chromatographic Conditions

UPLC measurements were carried out using an Acquity BEH Shield, RP18, 100 × 2.1 mm i.d., 1.7 µm column (Waters) operated at 35°C with gradient elution at 0.3 mL min^−1^ using mobile phase containing a gradient mixture of solvents A and B; UV absorbance at 223 nm; injection volume 5 µL. The LC gradient program was set as: time (min)/% mobile phase A/% mobile phase B: 0.01/40/60, 10/40/60, 13/10/90, 20/0/100, 35/0/100, 36/40/60, and 40/40/60. Water and acetonitrile (50:50 v/v) were used as diluent for sample preparation.

### Preparation of the Standard and System Suitability Solution

A stock solution of CH (220 µg mL^−1^) was prepared by dissolving an appropriate amount in diluent. Working solution was prepared from the above stock solution for related substances determination (1.1 µg mL^−1^ of CH) in diluent. Also, the impurity stock solutions (100 µg mL^−1^) were prepared in diluent.

### Preparation of the Sample Solution

Twenty tablets were weighed and the average weight of the tablet was calculated. Tablet powder equivalent to 27.5 mg of the active pharmaceutical ingredient (CH) was transferred into a 50-ml volumetric flask. To this 30 ml of diluent was added and it was sonicated for 30 minutes with intermediate shaking. The solution was then diluted to 50 ml with diluent and centrifuged at 3000 rpm for 10 min. The supernatant (550 µg mL^−1^ of CH) was collected, filtered through a 0.22 µ filter, and used as sample solution.

## Method Validation

The method was validated for specificity, linearity, precision, accuracy, robustness, and ruggedness, according to ICH guidelines [20].

### System Suitability

System suitability parameters were performed to verify the system performance. System precision was determined on six replicate injections of the standard preparation. All the important characteristics, including the relative standard deviation, peak tailing, and theoretical plate number, were measured. All of these system suitability parameters covered the system, method, and column performance.

### Specificity

Stress studies were performed at an initial concentration of 550 µg mL^−1^ of CH in the active pharmaceutical ingredients (API) and formulated sample to provide the stability-indicating property and specificity of the method. Intentional degradation was attempted by the various stress conditions i.e., acid (5 N HCl for 6 hours at 80°C), base (5 N NaOH for 6 hours at 80°C), oxidation (3% peroxide for 48 hours at 25ºC), water (refluxed for 6 hours at 80°C), heat (exposed at 105°C for 66 h), humidity (exposed to 90% RH for 7 days), and photolytic stress (1.2 million lux hours followed by 200 watt hours m^-2^).

### Precision

The precision for the determination of the impurities was checked by injecting six individual preparations of the CH (550 µg mL^−1^) test preparation spiked with 1.1 μg mL^−1^ of all the impurities and calculating the % RSD of each impurity. The intermediate precision of the method was also evaluated using different analysts and a different instrument in the same laboratory on a different day. Also, the precision study was performed by spiking 1.1 μg mL^−1^ of CH on a placebo as per test preparation.

### Limit of Detection (LOD) and Limit of Quantification (LOQ)

The LOD and LOQ for the CH impurities were estimated, the precision and accuracy were also determined at the LOQ level, and the % RSD and recovery were calculated for the peak area for each impurity and for CH.

### Linearity

Linearity solutions were prepared from the stock solutions at six concentration levels from the LOQ to 0.3% of the analyte concentration. The peak area versus concentration data were subjected to least-squares linear regression analysis. The calibration curve was drawn by plotting impurity areas against the concentration expressed in µg mL^−1^.

### Accuracy

The accuracy of an analytical procedure expresses the closeness of agreement between the true value and the observed value. The accuracy of the method was demonstrated at four different concentration levels in triplicate. The analysis was carried out by spiking all of the impurities on the formulation sample at the LOQ, 0.1, 0.2, and 0.3% of the CH concentration (550 µg mL^−1^). Also, the accuracy study was performed by spiking CH on the placebo at the above-mentioned levels. The percentage mean recoveries at each level for all the impurities and CH were calculated.

### Robustness

To determine the robustness of the developed method, the experimental conditions were deliberately changed and the resolution between CH & its impurities, and tailing factor and theoretical plates of the CH peak were evaluated.

To study the effect of the flow rate on the developed method, it was changed from 0.3 mL min^−1^ to 0.25 and 0.35 mL min^−1^. The effect of column temperature on the developed method was studied at 30 and 40°C (instead of 35°C). The effect of pH was studied by varying ± 0.2 pH units (i.e. 6.4 and 6.8) and the mobile phase composition was changed ±10% from the initial composition. In all of the above varied conditions, the component of the mobile phase was held constant.

### Stability of the Sample Solution and Stability of the Mobile Phase

CH-spiked samples (impurities spiked at 0.2% of the analyte concentration) were prepared in the diluent and the test solutions were left at room temperature. The spiked samples were injected at time intervals of 0, 24, and 48 hrs. The impurity content was calculated and the consistency in the % of each impurity at each interval was checked. The prepared mobile phase was kept constant during the study period. The mobile phase study was demonstrated by injecting the freshly prepared sample solution at different time intervals (0–2 days).

## Results and Discussion

### Optimization of Chromatographic Conditions

The main objective was to develop an RP-LC method for the determination of impurities in the cinacalcet pharmaceutical dosage form in a single run. The developed method should be accurate, reproducible, robust, stability-indicating, linear, free of interference from other formulation excipients, and convenient enough for routine use in quality control laboratories.

**Fig. 2:**
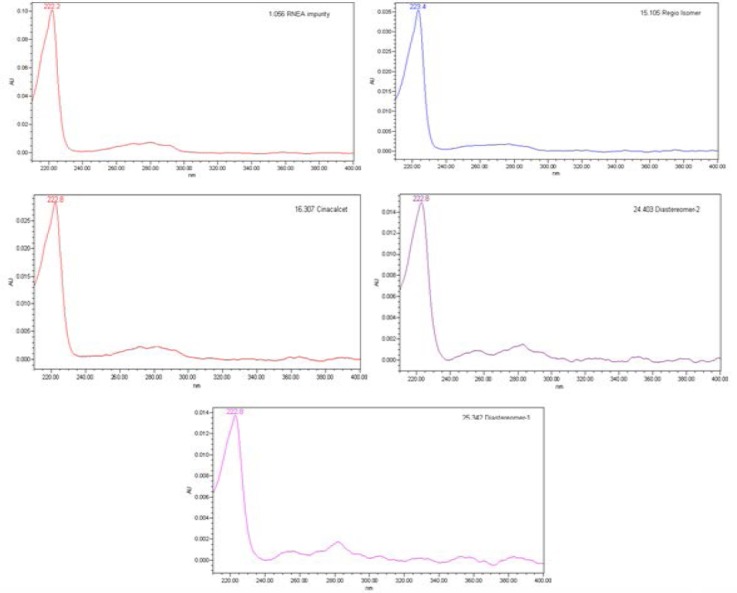
Overlay spectra of cinacalcet HCl and its impurities

Individual stock solutions of CH and its impurities of same concentrations were scanned in a photodiode array detector in the range of 200 to 400 nm and the spectra of each component was checked. From the spectra, all the impurities had an absorbance maximum at about 223 nm. Hence, 223 nm was selected for the estimation of CH impurities. The typical spectra for CH and its impurities are shown in Fig. 2. The mass spectra of diastereomer-1 and 2 are presented in Figs 3a & 3b.

**Fig. 3a:**
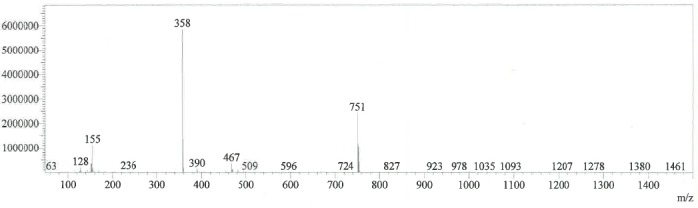
Mass spectra of diastereomer-1

**Fig. 3b:**
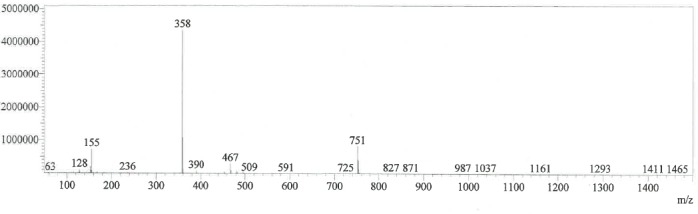
Mass spectra of diastereomer-2

The CH sample preparation (550 µg mL^−1^) spiked with all the impurities (1.1 µg mL^−1^) and placebo preparations were subjected to separation by RP-HPLC. Initially, the separation of all peaks was studied using a 0.02 M potassium dihydrogen orthophosphate of pH 6.6 buffer with gradient elution by using C8 and C18 columns. Better separation was observed in the C18 column compared to the C8 column. In the C18 column, all the peaks were well–separated, but the run time was too long, lasting about 70 minutes. Shorter column lengths were tried along with a higher acetonitrile composition, but there was no improvement in the run time. To achieve a shorter run time, HPLC was replaced with UPLC by using the Acquity BEH Shield RP18 100 × 2.1 mm, 1.7 micron column.

Different mobile phase compositions were tried along with different gradient programs. Finally, the chromatographic separation was achieved on an Acquity BEH Shield, RP18, 100 × 2.1 mm i.d., 1.7 µm column (Waters) operated at 35°C with gradient elution at 0.3 mL min^−1^ using a mobile phase buffer as 0.02 M potassium dihydrogen orthophosphate of pH 6.6, containing 1 mL of triethylamine (pH-adjusted with dilute orthophosphoric acid solution); UV absorbance at 223 nm; injection volume 5 µL. Mobile phase A consisted of a pH 6.6 phosphate buffer and acetonitrile (90:10 v/v); mobile phase B consisted of a pH 6.6 phosphate buffer and acetonitrile (45:55 v/v). The LC gradient program was set as: time (min)/% mobile phase A/% mobile phase B: 0.01/40/60, 10/40/60, 13/10/90, 20/0/100, 35/0/100, 36/40/60, and 40/40/60. All the impurities were well-separated with a resolution greater than 2, the typical retention times of CH, RNEA, regioisomer, diastereomer isomer-1, and diastereomer isomer-2 were 15.495 min, 1.041 min, 14.595 min, 24.575 min, and 23.820 min, respectively. No chromatographic interference due to the blank (diluent) and other excipients (placebo) at the retention times of CH and all of the impurities were observed. The resolution between the placebo and RNEA peak was found to be 2.9. Water and acetonitrile (50:50 v/v) was used as diluent for the sample preparation.

### Response Factor

The measurement of the response factor for each impurity determination is important when the calculations are being made on a relative percent basis. Hence, the authentic sample of the known impurities and CH were dissolved in the diluent and injected, then responses were calculated. The observed relative response factor values of RNEA, regioisomer, diastereomer isomer-1, and diastereomer isomer-2 were 1.79, 0.99, 0.89, and 0.88, respectively.

## Method Validation

After the development of the method, it was subject to method validation as per ICH guidelines [20]. The method was validated to demonstrate that it is suitable for its intended purpose by the standard procedure to evaluate adequate validation characteristics (system suitability, specificity, accuracy, precision, linearity, robustness, ruggedness, solution stability, LOD and LOQ, and stability-indicating capability).

### System Suitability

Results from the system suitability study are given in Table 1.

**Tab. 1:**
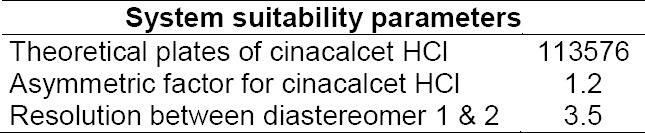
System suitability results

The typical overlay chromatogram of the blank, standard, placebo, and spiked test is shown in Fig. 4a & Fig. 4b.

**Fig. 4a:**
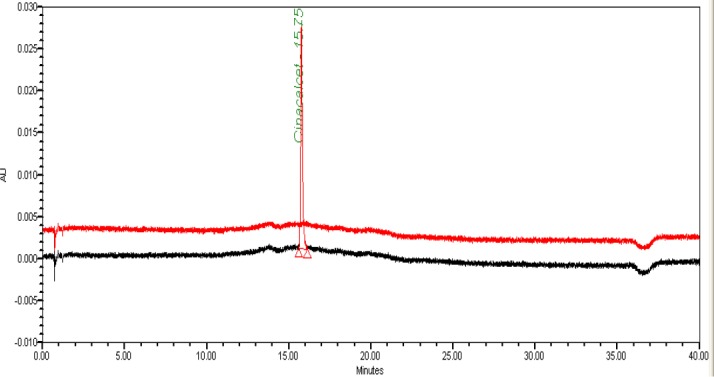
UPLC overlay chromatogram of the blank and standard solution

**Fig. 4b:**
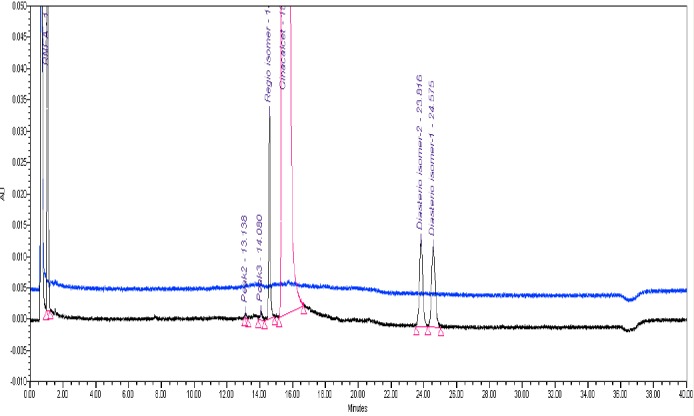
UPLC overlay chromatogram of the placebo and spiked sample

### Specificity – Stress Study

All forced degradation samples were analyzed with the aforementioned UPLC conditions using a PDA detector to monitor the homogeneity and purity of the CH peak and its related impurities. The individual impurities, placebo, and CH were verified and proved to be non-interfering with each other, thus proving the specificity of the method.

**Fig. 5:**
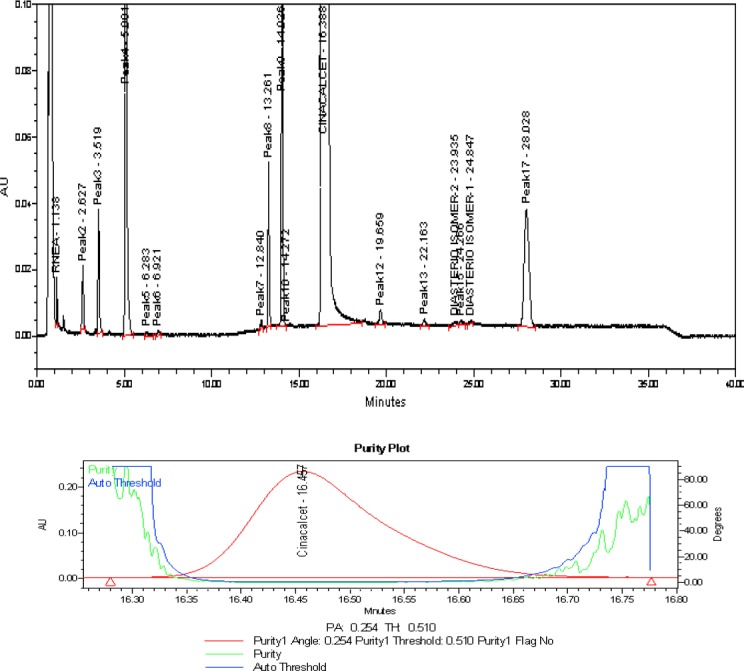
Typical chromatogram and purity plot of the peroxide-stressed sample

Figure 4b shows that there is no interference at the RT (retention time) of CH and all known impurities from the other excipients. There are two unknown impurities that were observed at the RT of 13.138 and 14.080 minutes for which the area % was found to be less than the reported threshold limit i.e., 0.1%. Degradation was not observed in the acid stress, base stress, water stress, heat stress, humidity stress, and photo stress studies. Significant degradation was observed in the peroxide stress study. It is interesting to note that all the peaks, due to degradation, were well-resolved from the peaks of CH and its impurities with a resolution of more than 2.5. Also, the resolution between the peroxide peak and RNEA peak was found to be 2.8. For all forced degradation samples, the purity angle (the weighted average of all spectral contrast angles calculated by comparing all spectra in the integrated peak against the peak apex spectrum) was found to be less than the threshold angle (the sum of the purity noise angle and solvent angle, the purity noise angles across the integrated peak) and there was no purity flag (the purity flag is an indication of spectral homogeneity, compares the purity angle with the purity threshold) for CH and its impurities. The verification of peak purity indicates that there is no interference from degradants, facilitating error-free quantification of CH impurities. The degraded sample solutions of CH were assayed against the qualified reference standard for the mass balance study. The mass balance of the stressed samples was found to be more than 96%. Thus, the method is considered to be “stability-indicating.” The specificity results and peroxide degradation chromatogram along with peak purity are shown Table 2 and Fig. 5.

**Tab. 2:**
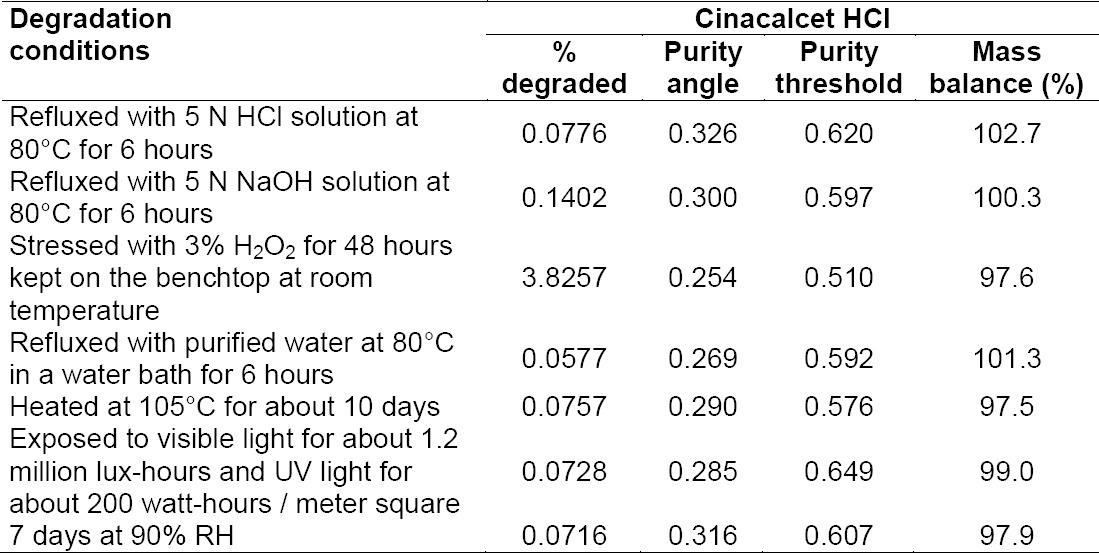
Forced degradation data for cinacalcet

### Precision

The % RSD for the individual % of CH and its impurities in the method precision study was within 2.1%. The results obtained in the intermediate precision study for the % RSD of the individual % of CH impurities were well within 2.2%, confirming the high precision of the method. The results are shown in Tables 3 & 4.

**Tab. 3:**
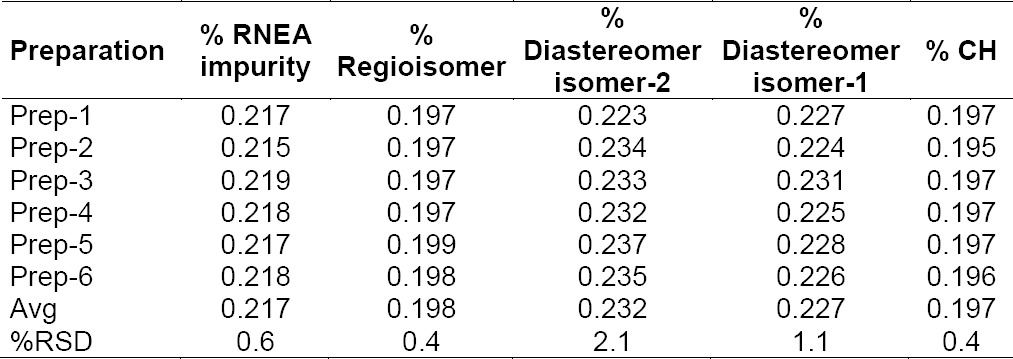
Precision of the method

**Tab. 4:**
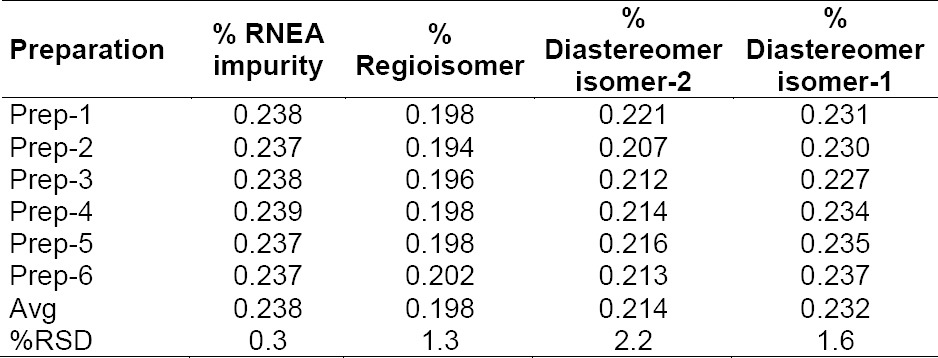
Intermediate precision of the method

### Limit of Detection and Quantification

LOD values were achieved at 0.036, 0.082, 0.164, 0.167, and 0.084 µg mL^−1^ for RNEA, regioisomer, diastereomer isomer-1, diastereomer isomer-2, and CH, respectively. LOQ values were achieved at 0.11, 0.22, 0.47, 0.45, and 0.22 µg mL^−1^ for RNEA, regioisomer, diastereomer isomer-1, diastereomer isomer-2, and CH, respectively. The % RSD of precision at the LOQ concentration for CH and its impurities were found to be below 5.4. The results of precision at the LOQ level are shown in Table 5.

**Tab. 5:**
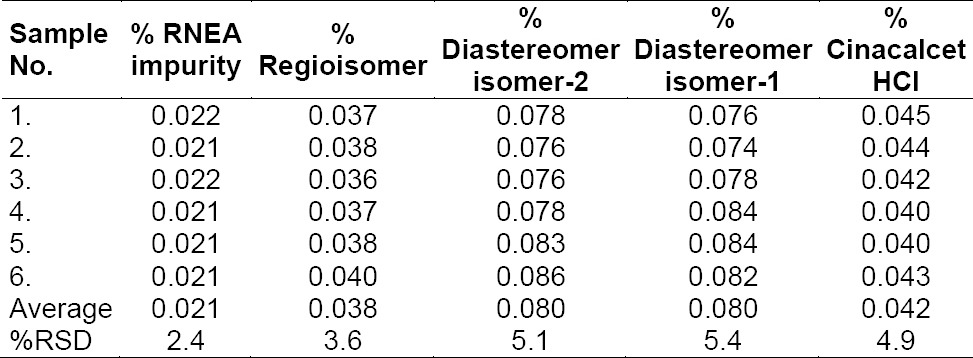
Table of results of precision at the limit of quantification

### Accuracy

The recovery of all four impurities and CH from the finished pharmaceutical dosage form ranged from 85.0% to 115.0%. The summary of % recovery for each individual impurity is mentioned in Table 6.

**Tab. 6:**
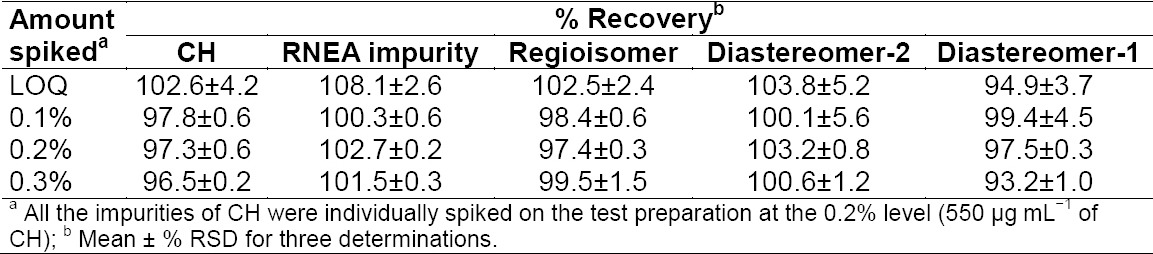
Accuracy of the method

### Linearity of Response

**Fig. 6:**
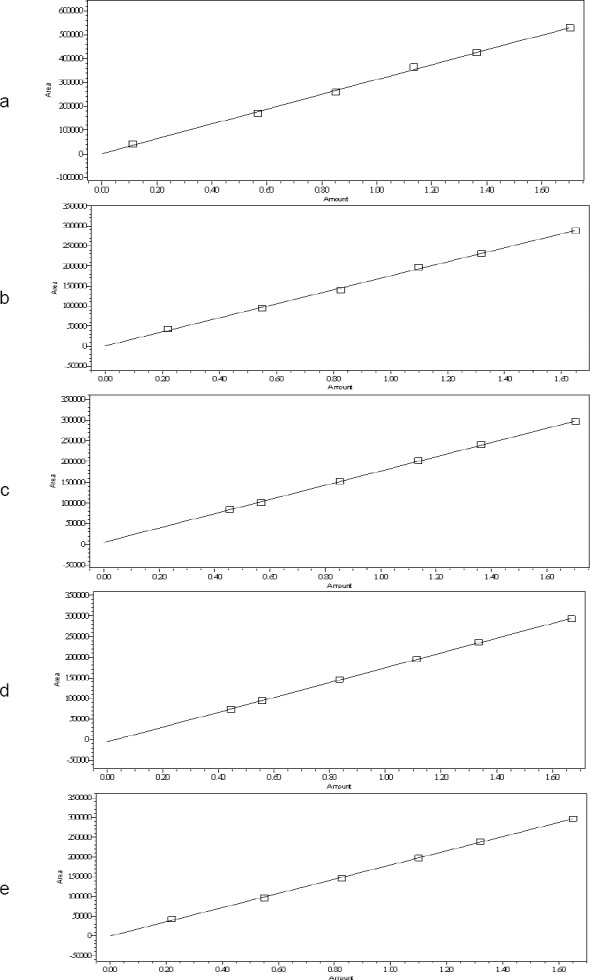
Linearity graph of the RNEA impurity (a), regioisomer (b), diastereomer-1 (c), diastereomer-2 (d), and cinacalcet HCl (e)

A linear calibration plot for CH and its impurities was obtained over the calibration range of the LOQ (0.11, 0.22, 0.47, 0.45, and 0.22 µg mL^−1^ for RNEA, regioisomer, diastereomer isomer-1, diastereomer isomer-2, and CH, respectively) to 1.7 μg mL^−1^ and the correlation coefficient was found to be about 0.999. The coefficient of correlation was found to be more than 0.997. The regression statistics are shown in Table 7. The linearity curve for all impurities is represented in Figs 6a, 6b, 6c, 6d, & 6e.

**Tab. 7:**
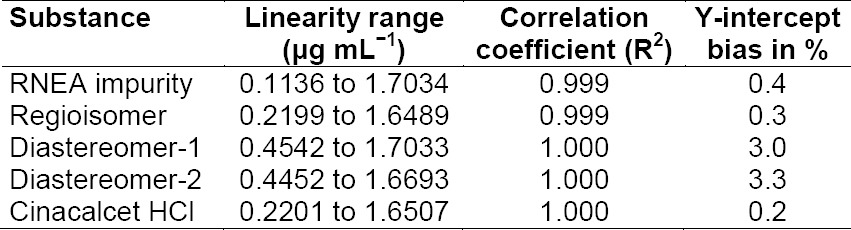
Table of results of linearity

### Robustness

No significant effect was observed on the system suitability parameters such as RSD, tailing factor, or the theoretical plates of CH when small, but deliberate changes were made to the chromatographic conditions. Thus, the method was found to be robust with respect to variability in the applied conditions.

### Stability of the Sample Solution and Stability of the Mobile Phase

No significant changes were observed in the content of CH impurities during the solution stability and mobile phase stability experiments when performed using the impurities method. The solution stability and mobile phase stability experiment data confirmed that the sample solution and mobile phases used during the impurity determination were stable for at least 48 h.

## Conclusion

The gradient UPLC method developed for the determination of CH impurities in both bulk drug and pharmaceutical dosage forms was precise, accurate, and specific. The method was validated as per ICH guidelines and found to be specific, precise, linear, accurate, rugged, and robust. The developed method can be used for the stability analysis of both CH API and formulated samples.
